# Intelligence, Correspondence, &c.

**Published:** 1829-07-01

**Authors:** 


### INTELLIGENCE, CORRESPONDENCE, &c.

### GIBRALTAR EPIDEMIC.

We hardly regret to learn that we laboured under a mistake when we adverted to the inactivity of our own Government, and the industry of that of France, in the investigation of the late Epidemic which ravaged Gibraltar. We have the best reason to believe that both the local Government of the Rock, and the general Government at home, have taken as prompt measures as were compatible with existing circumstances, to inquire into the causes, nature, and probable prevention of the Gibraltar Fever.

### APOTHECARIES' HALL.

It gives us great satisfaction to be able to state publicly, what indeed we have been able to state privately for some time past, that the Court of Examiners, of the above-mentioned Company, *do* admit of lectures *attended by pupils during their apprenticeship,* as proofs apparent of professional education—thus, in fact, doing away with much of the inconvenience attendant on the apprenticeship Act. Mr. Wakley has seized on a misrepresentation (for misrepresentations are his only props) in order to give a little excitement to a stale story. But the day of medical calumny, like the "no popery" cry, is past!

### *Literary Intelligence.*

Dr. KENNEDY has, in a state of readiness for the press, a Work which will form three volumes in 8vo. and be entitled, *A History of the Medical Sciences, Biographical and Philosophical, containing an Account of the Persons and Writings that have conduced to the Improvement of Physick, from its origin in* BRITAIN *to the End of the Eighteenth Century.* This Work naturally admits of a twofold arrangement—PART I. consists of more than One Thousand Articles, each of which is subdivided into two sections; a *Biographical,* wherein the circumstances of its subject, both personal and professional, are concisely represented; and a *Philosophical,* which describes the most important Discoveries, Principles and Doctrines propounded in the same Individuals' Writings;—into these Sections, Disquisitive and Analytical Illustrations are occasionally introduced. PART II. includes an Alphabetical List of all Cases, Essays, and Books having relation to the Progress or Applications of Medical Knowledge; it also comprehends anonymous Treatises, Translations, and Papers contributed to Periodical Journals and the Transactions of Societies, by Writers whose History could not be obtained. Each volume will be provided with a minute and copious Index, so as to make the Work a complete system of reference to the elements of our British Medical literature and philosophy.

In answer to MEDICUS and several other inquirers, we have to observe that, all *accepted* articles, in the Review department, are paid for at a reasonable rate; but we cannot foreclose. We prefer the articles being sent with a sealed name and motto.

N. B. An article (in the shape of a review) on TEMPERAMENT, and its influence on health, disease, and remedial treatment, is wanted. The author will have the option of being paid for the article in the usual manner, in which case, the name of the writer must, of course, remain concealed—or the successful candidate will be awarded a prize of a complete set of this Journal bound, in eleven volumes, and the name of the author will be published three months after the insertion of the article. The paper is to be sent with sealed name and motto, as above-mentioned.

